# Human papillomavirus 16 E2-, E6- and E7-specific T-cell responses in children and their mothers who developed incident cervical intraepithelial neoplasia during a 14-year follow-up of the Finnish Family HPV cohort

**DOI:** 10.1186/1479-5876-12-44

**Published:** 2014-02-13

**Authors:** Hanna-Mari Koskimaa, Anna E Paaso, Marij JP Welters, Seija E Grénman, Kari J Syrjänen, Sjoerd H van der Burg, Stina M Syrjänen

**Affiliations:** 1 Medicity Research Laboratory and Department of Oral Pathology, Institute of Dentistry, Faculty of Medicine, University of Turku, Turku, Finland; 2 Department of Obstetrics and Gynaecology, Turku University Hospital, Turku, Finland; 3 Department of Oncology and Radiotherapy, Turku University Hospital, Turku, Finland; 4 Teaching and Research Institute, Barretos Cancer Hospital, Barretos-SP, Brazil; 5 Department of Clinical Oncology, Leiden University Medical Center, Leiden, The Netherlands

**Keywords:** HPV16, T-cell immunity, Cytokines, Children, Mothers

## Abstract

**Background:**

Human papillomavirus (HPV) infection has traditionally been regarded as a sexually transmitted disease (STD), but recent evidence implicates that an infected mother can transmit HPV to her newborn during pregnancy, at delivery, perinatal period or later. Given the lack of any studies on HPV-specific immune responses in children, we conducted HPV16-specific cell-mediated immune (CMI) monitoring of the mother-child pairs with known oral and genital HPV follow-up (FU) data since the delivery. In the Finnish Family HPV Study, 10 out of 331 mothers developed incident cervical intraepithelial neoplasia (CIN) during their 14-year FU. Our hypothesis according to the common dogma is that there is no HPV16 specific immune response in offspring of the CIN mother as she/he has not started the sexual life yet.

**Methods:**

We used overlapping 30–35 mer peptides covering the entire HPV16 E2, E6 and E7 protein sequences. Assays for lymphocyte proliferation capacity, cytokine production and HPV16-specific Foxp3 + CD25 + CD4+ regulatory T-cells were performed.

**Results:**

HPV16-specific proliferative T-cell responses were broader in children than in their mothers. Nine of 10 children had responses against both E2 peptide pools compared to only 4 of the 10 mothers. Six of the 10 children and only 2 mothers displayed reactivity to E6 and/or E7. The cytokine levels of IL-2 (p = 0.023) and IL-5 (p = 0.028) induced by all peptide pools, were also higher among children than their mothers. The children of the mothers with incident CIN3 had significantly higher IFN-γ (p = 0.032) and TNF-α (p = 0.008) levels than other children.

**Conclusions:**

Our study is the first to show that also children could have HPV-specific immunity. These data indicate that the children have circulating HPV16-specific memory T-cells which might have been induced by previous HPV16 exposure or ongoing HPV 16 infection.

## Background

Persistent high-risk (HR) human papillomavirus (HPV) infection is the most important risk factor for cervical cancer (CC). Of the HR-HPV genotypes, HPV16 is the most prevalent, present in 50% of all CC cases worldwide [1,2]. It has been estimated that over 80% of women will encounter genital HPV infection during their life-time [3] but only a minority of these infections will ever progress to malignancy [4,5]. Based on recent meta-analysis, around 291 million women worldwide are carriers of HPV DNA, of whom 32% are infected with HPV16 or HPV18, or both [6].

HPV-induced immunity is crucial for clearance of the infection. However, HPV is known to have several mechanisms to evade the host immune system, as recently discussed [7-12]. HPV replication is linked with the differentiation of keratinocytes in a way that inhibits virus detection by the host. In productive HPV infections, viral particles are released from the dying keratinocytes on the epithelial surface. Thus, there is neither cytolysis inducing inflammation nor pro-inflammatory cytokines activating macrophages or Langerhans cells (LC) in infected epithelia [8]. According to current concepts, there is no blood-borne stage in the life cycle of HPV and the viral early (E) proteins are expressed only at low levels in the nucleus of an infected cell. Oncoproteins E5, E6 and E7 of the HR-HPV can modulate antigen presentation by impairing the assembly of peptide-MHC complexes, and block the anti-viral responses at the stage of cell entry and virus presentation, by modifying the production and functions of cytokines such as interferons [7].

Until now, most research on HPV immunity has been focused on cervical HPV infections in women. HPV immunity is based on circulating HPV-specific CD4+ T helper (Th) and CD8+ cytotoxic T lymphocytes (CTLs) of the adaptive, cell mediated immune (CMI) system [12]. These cells can be targeted to the E and late (L) HPV proteins and migrate to the site where the viral proteins are presented [12]. In healthy subjects, circulating CD4+ and CD8+ T cells specific for HPV16 E antigens are frequently detected [13,14], while HPV-induced cervical intraepithelial neoplasia (CIN) or CC are characterized by weak HPV E antigen-specific T-cell immunity, and these merely CD4+ T cells have an impaired capacity to produce Th type 1 (Th1) and type 2 (Th2) cytokines [7,12,13,15-17]. In contrast to effector cells, regulatory T cells (Tregs) are known to suppress virus-specific immunity [18]. HPV16-specific Foxp3+ CD4+ regulatory T-cells have been associated with immunosuppressive responses as they are capable of inhibiting the proliferation of naïve CD4+ T-cells and Th1-cells and their cytokine production [17,19,20].

HPV infection has been traditionally considered as a sexually transmitted disease (STD). However, recent meta-analysis confirmed that newborns of the mothers with genital HPV infection had 33% higher risk to acquire HPV infection than those of HPV-negative mothers [21,22]. Vertical transmission from an infected mother to her newborn can occur during pregnancy via infected placenta, cord blood, ascending cervical infection or through an infected birth canal at delivery [23,24]. Horizontal HPV transmission might occur for instance via breast-feeding, from sibling via kissing or friends via digital contacts [22,25]. Persistent oral HPV in newborn has been well documented but with highly variable frequencies from 1% up to 83% [26-29]. We have recently shown that 18% of the oral samples of the newborns and 16% of the cervical samples of mothers tested HPV DNA-positive. The HPV genotype profile of the mother-newborn pairs was almost identical at delivery but lost this similarity during the first 2 months [24].

The Finnish Family HPV study was designed in 1998 to elucidate the dynamics of HPV transmission between the family members [30,31]. In the present study, we analyzed the HPV16-specific CMI responses among the women who developed an incident CIN. HPV16-specific immunity was related to their known oral and genital HPV DNA status and HPV serology during the FU. Of special interest was to assess whether or not their children have developed HPV16-specific CMI reactivity at the same time point. Notable is that none of these children has been HPV vaccinated or started his/her sexual debut.

## Methods

### Mothers and their children

The Finnish HPV Family Study is a longitudinal cohort study conducted at the Department of Oral Pathology, Institute of Dentistry, University of Turku and Department of Obstetrics and Gynecology, Turku University Central Hospital. The study plan was approved by the Research Ethics Committee of Turku University Hospital (#3/1998 and 45/180/2010). Originally, 329 pregnant women at their 3rd trimester pregnancy and all their newborns (n = 331; included two twins) were enrolled in the study between 1998 and 2001, as described previously [29-31]. During the FU until present, 12 women developed an incident CIN, as recently reported [32]. These women and their children were recalled for blood sampling. Written informed consent was obtained from 10 of these women, with mean age of 37.0 years and 12.2 years of their children, respectively. Three of the children were girls and seven were boys. None of these children had had any sexual contacts or received prophylactic HPV vaccination before or during study onset (Characteristics of the mothers and their children are summarized in Additional file 1: Table S1). As a negative control group three healthy women who remained HPV negative during FU were included with their children (in total two girls and one boy). The mothers and their children were at the same age as those in the study group. HPV detection methods as well as the antibody screening against the major capsid protein L1 of HPV 16 have been detailed in previous reports [22,24,32,33].

The authors acknowledge the concept of the Minimal Information About T cell Assays (MIATA) framework, which was recently published [34,35]. Therefore, detailed information is provided as structured in the proposed 5 modules by MIATA: the sample, the assay, the data acquisition, the data analysis and the laboratory environment in which the human T-cell assays were performed.

### Blood samples

Venous blood samples were collected from mothers (74 mL) and children (54 mL) in sodium-heparin collection tubes and peripheral blood mononuclear cells (PBMCs) were isolated within three hours by centrifugation over Ficoll-Hypaque gradient (GE Healthcare Life Sciences, Uppsala, Sweden) and washed with PBS. Viable and dead cells were counted using trypan blue staining. For determining the proliferative capacity of HPV16–specific T cells by short–term lymphocyte stimulation test (LST), ~10×10^6^ PBMCs were used and the remaining cells were frozen in 80% Fetal Bovine Serum (FBS, Biowest, EU quality) and 20% DMSO (Merck, Darmstadt, Germany) (10 million PBMCs per mL per vial) using Nalgene™ Cryo 1°C Freezing container, which ensures a −1°C/min rate of cooling, at −70°C, and after 1–2 days stored the cryopreserved PBMCs in liquid nitrogen until further use. The median time from sample collection until beginning of LST or cryopreservation was 3 hours. From 9 mL of blood collected into clotting tube the serum was isolated by centrifugation for 7 minutes at 1000 *g*. Obtained autologous serum was used for short-term T-cell proliferation assay as described previously [36,37].

### HPV16 peptides

Panels of overlapping 30–35 mer peptides with HPV16 E2, E6, and E7 protein sequences were synthesized by solid phase peptide synthesis (SPPS) method with >95% purity (ChinaPeptides Co. Shanghai, China), with a 14 (for 30-mer) or 15 (for 35-mer) amino acid (aa) overlap. Two pools of E2 peptides (E2.1 and E2.2) consisted of 12 or 11 (30-mer) peptides, respectively. Four pools of E6 and two pools of E7 peptides (E6.1-E6.4 and E7.1 and E7.2) consisted of two 32-mer or 35-mer peptides, respectively. The peptides are detailed in Table 1. Peptide quality was tested by mass spectrum and high-performance liquid chromatography (HPLC). Lyophilized peptides were dissolved in distilled water or in water with 10% DMSO for proper dissolution. Peptides were stored at −20°C with the final concentration of 1 mg/mL. Eight peptide pools were used to determine the proliferative capacity of HPV16-specific T-cells, for cytokine production analysis and detection of the HPV16-specific Foxp3+ regulatory T-cells. Memory response mix (MRM) stock solution (50×), consisting of tetanus toxoid, 0.75 fL/mL (Statens Serum Institut, Copenhagen, Denmark), Tuberculin PPD, 5 μg/mL (Statens Serum Institut), and Candida albicans, 0.015% (Greer Laboratories, Lenoir, USA) was used as a positive control for the proliferation assays and cytokine production capacity of the PBMCs [13].

**Table 1:** HPV16 peptides among the 8 peptide pools

**HPV16 E2**					
**Pool**	**Peptide**	**Amino acids**	**Pool**	**Peptide**	**Amino acids**
**E2.1**	E2-1	1-30	**E2.2**	E2.13	181-210
	E2-2	16-45		E2.14	196-225
	E2-3	31-60		E2.15	211-240
	E2-4	46-75		E2.16	226-255
	E2-5	61-90		E2.17	241-270
	E2-6	76-105		E2.18	256-285
	E2-7	91-120		E2.19	271-300
	E2-8	106-135		E2.20	286-315
	E2-9	121-150		E2.21	301-330
	E2-10	136-165		E2.22	316-345
	E2-11	151-180		E2.23	331-365
	E2-12	166-195			
**HPV16 E6**		**HPV16 E7**			
**Pool**	**Peptide**	**Amino acids**	**Pool**	**Peptide**	**Amino acids**
**E6.1**	E6.1	1-32	**E7.1**	E7.1	1-35
	E6.2	19-50		E7.2	22-56
**E6.2**	E6.3	37-68	**E7.2**	E7.3	43-77
	E6.4	55-86		E7.4	64-98
**E6.3**	E6.5	73-104			
	E6.7	91-122			
**E6.4**	E6.8	109-140			
	E6.9	127-158			

The original protein sequences were derived from NCBI Protein database: http://www.ncbi.nlm.nih.gov/protein. Accession numbers: AAA46941.1 (HPV16 E2), 4927720 (HPV16 E6), and 739162 (HPV16 E7).

### Determination of the proliferative capacity of HPV16–specific T cells by short–term lymphocyte stimulation test (LST)

The freshly isolated PBMCs with a density of 1.5 × 10^5^ cells per well were seeded into U-bottomed 96-wells microtiter plate (Nunc, Roskilde, Denmark). Eight replicative wells were used for each peptide pool. The PBMCs were cultured in IMDM (Gibco, Life Technologies, Belgium) containing 10% autologous serum and the indicated peptide pool at a final concentration of 5 μg/mL per peptide. PBMCs cultured with MRM were used as a positive control (10 μl/well of 4xMRM) and with no antigen (medium-only) as a background control. After 6 days of culturing the supernatants of all eight replicative wells were collected and pooled for cytokine analysis. A compensatory amount of IMDM supplied with 0.5 μCi [^3^H]-Thymidine (PerkinElmer, Turku, Finland) per well was added. After 18 hours of incubation, the cells were harvested into Unifilter plates (PerkinElmer) using the FilterMateTM Cell Harvester (PerkinElmer). Subsequently, the filter plates were dried and counted on the 1450 MicroBeta + counter (PerkinElmer). The cut-off value for counts per minute (CPM) values was determined by the average plus 3 × SD of the eight medium-only control wells. Stimulation index (SI) was calculated as the average of tested eight wells divided by the average of the medium-only control wells. The proliferative response was defined positive if the CPM values of at least six of the eight wells were above the cut-off value and if the SI was ≥3 [13,14].

### Analysis of cytokine production

The supernatants collected from LST at day 6 were analyzed using Cytometric Bead Array (CBA) human enhanced sensitivity flex set system (BD Biosciences, Temse, Belgium) according to the manufacturer’s instructions. In this array, the levels of IFN-γ, TNF-α, IL-2, IL-5, IL-10, and IL-17A were determined. The detection limits for the cytokines were based on standard curves complying with the limit of 274 fg/mL described by the manufacturer. The positive antigen-induced cytokine production was defined as a cytokine concentration > 2× the concentration of the medium-only control [38]. Cytokines were not analyzed in the children of the negative control group.

### Identification of HPV16 –specific CD4 + CD25 + Foxp3+ regulatory T cells

Frozen PBMCs were thawed and seeded into 24-wells plate (1.0 × 10^6^ cells/well). The cells were cultured in IMDM containing 10% Human AB serum (Sigma-Aldrich, San Louis, USA) and the indicated peptide pools (Table 1) at a final concentration of 5 μg/mL per peptide. PBMCs cultured with MRM were used as a positive control (80 μl/well of 4xMRM) and without antigen (medium-only) were used as a background control. After 7 days of culturing, the cells were harvested, washed with 0.5% BSA in PBS and stained first with surface markers CD25 (1:25) (Anti-CD25 FITC, clone M-A251, BD Pharmingen, San Diego, CA), CD4 (1:100) (Anti-CD4-APC, clone RPA-T4, BD Pharmingen), CD8 (1:30) (Anti-CD8 PerCP-Cy5.5; clone SK1, BD Pharmingen). Subsequently, the cells were fixed and permeabilized using intra-nuclear staining buffer set (FOXP3 Fix/Perm buffer set, Biolegend, San Diego, CA) according to manufacturer’s instructions. Before intracellular staining with Foxp3 (PE anti-human FOXP3, Biolegend) or isotype control (PE Mouse IgG1, κ Isotype Ctrl, Biolegend), blocking was done with 2% FCS. All staining procedures were done on ice. After staining, the cells were washed, resuspended in PBS and measured by the flow cytometer BD FACSCalibur (BD Bioscience). Analysis was performed by using Flowing Software, version 2.5.0 (Cell Imaging Core, Turku Centre for Biotechnology, Turku, Finland). The fluorescent intensity of MRM-stimulated and medium-only control cells was used to set the gates for the other samples. An antigen-induced alteration in the population percentage was defined as a change of at least 2× the corresponding percentage in the medium-only control [36,38]. HPV16 –specific CD4 + CD25 + Foxp3+ regulatory T cells were not determined in the children of the HPV negative control group.

### Statistical analysis

All statistical analyses were run using IBM SPSS® (IBM, Inc., New York, USA) software package (IBM SPSS Statistics for Windows, version 20.0.0.1). Frequency tables were analysed using the χ2-test, with the likelihood ratio or Fisher’s exact test for categorical variables. Differences in the means of continuous variables were analysed using non-parametric (Mann-Whitney or Kruskal-Wallis) tests for two- and multiple independent samples, respectively. Paired-samples test (Wilcoxon) was used to analyse the response levels in mother-child pairs. All statistical tests were two-sided and declared significant at p-value ≤0.05.

### Laboratory environment

The laboratory of the Oral Pathology at the Institute of Dentistry, Faculty of Medicine, University of Turku, Turku, Finland, is a research laboratory where the T-cell assays are performed according to SOPs, including the predefined criteria for positive responses. All the methods needed for the CMI studies were transferred from the laboratory of van der Burg under his and Dr. Welter’s close guidance and quality control.

## Results

### Woman with incident CIN and their children

Additional file 1: Table S1 summarizes the medical history of the mothers’ cervical lesions, oral and genital HPV status and HPV-specific serology, as well as the oral HPV DNA status and HPV serology of their children, followed since birth until present. Among the women with incident CIN, five women had CIN3, three had CIN2 and two developed CIN1. Long-term persistence of HPV16 preceded all incident CIN2 and CIN3 lesions. Five women (IDs 1A, 2A, 3A, 6A, and 8A) tested also occasionally HPV16-positive in their oral mucosal samples (data not shown), and seven women (IDs 1A, 2A, 3A, 4A, 6A, 7A and 10A) had HPV16 antibodies detectable in sera at different time points. Six children (IDs 2B, 3B, 5B, 6B, 8B, 10B) tested also occasionally HPV positive in their oral samples, of whom two children were HPV16 positive (IDs 8B and 10B).

### Children have better HPV16-specific proliferative T-cell response than their mothers

**Figure 1:**
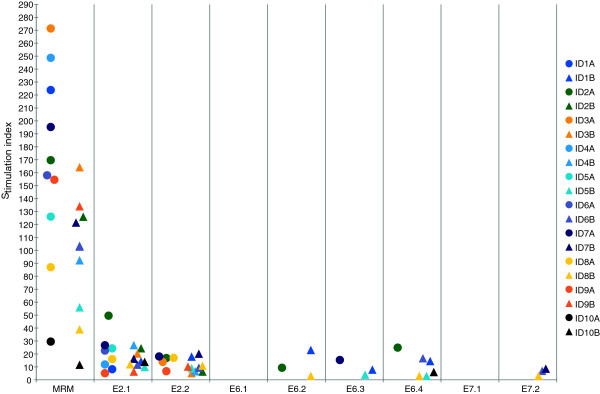
**Proliferative T-cell responses.** Proliferative T-cell responses against peptide pools of HPV16 E2, E6 and E7 of the ten mothers and their children. (●) represents stimulation indexes of the mothers and (∆) stimulation indexes of their children. The mother and her child are labelled by the same color. Only responses considered positive are shown. MRM = memory response mix.

PBMCs of all ten mothers and children showed proliferative response against MRM (Figure 1). The MRM responses in mothers were stronger than in children, although the range of SI was wide both in mothers (30–271) and their children (12–164). HPV16-specific proliferative T-cell response was found in 9 out of 10 mothers and in all the ten children at least to one of the peptide pools of E2, E6 and E7 (Figure 1). Nine mothers had reactivity to the peptide pool E2.1 (aa 1–195) and four of them had reactivity also to the pool E2.2 (aa 181–365). Only two mothers had response to E6 peptides; one to peptide pools E6.2 and E6.4 (ID2A) and the other to peptide pool E6.3 (ID7A). No HPV16 E7-specific response was found in any of the mothers.

**Figure 2:**
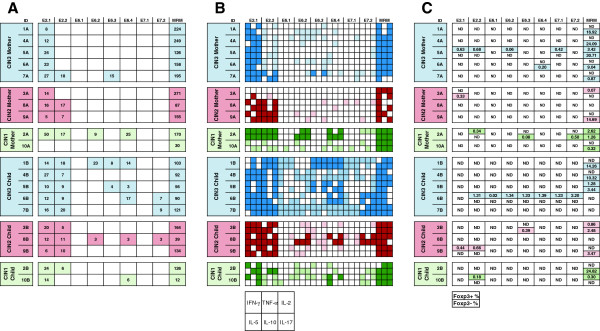
**Results of LST, cytokine and Foxp3 assays among CIN1, CIN2 and CIN3 mothers and their children. A)** HPV16 specific lymphocyte stimulation test (LST) results. The mothers and their children are grouped according to the mothers CIN. Only positive responses are marked with colored box and stimulation indexes are given in the boxes under the corresponding peptide pools. The blue color is used for the mothers with CIN3 and their children, the red color for the mothers with CIN2 and their children and the green color represent mothers with CIN1 and their children (the same color codes are used also in Figure 2B and 2C). Memory response mix (MRM) was used as a positive control for LST assay. **B)** Cytokine assay results. Supernatants from the LST were analyzed for the presence of cytokines IFN-γ, TNF-α, IL-2, IL-5, IL-10, and IL-17. One square in a box with 6 squares represents the production of the cytokine type as given in the index box lowest in the figure. Cytokines found in those supernatants from wells with positive proliferative responses are marked with dark colors. Lighter colors are used when the corresponding LST result was negative. **C)** Foxp3 assay results. The box, when marked is divided into two parts. The upper value presents the percentages of (CD4 + CD25+) Foxp3–positive cells while the lower value presents the (CD4 + CD25+) Foxp3-negative cells after subtraction of the medium-only control value. Only positive (upregulation) responses are shown. Upregulation of Foxp3+/− cells is defined as at least twice the percentages of those in the medium-only control. Foxp3 = forkhead box P3, ND = not detectable.

One of the mothers had no response to any of the peptide pools (ID10A). She had a persistent HPV16 infection detectable for at least 72 months and eventually developed a CIN1 lesion with an HPV16/66 double infection. She had HPV16 antibodies detectable only once; one year after study entry, despite persisting HPV16. In addition, she showed the lowest proliferative T-cell response to the common recall antigens in the MRM (Figure 2A) which might indicate an impaired overall CMI response. All but one (ID7A) of the mothers who developed CIN2 or CIN3 displayed HPV16-specific proliferative T-cell responses to E2.1 but did not respond to E6 and/or E7. This particular mother (ID7A) responded to E6.3 (Figure 2A), confirming our previous data that women with HSIL do not properly respond to the oncoproteins E6 and E7 [17,39].

All children had HPV16-specific proliferative T-cell response against the peptide pool E2.1, and 9/10 also responded to the pool E2.2 (Figure 2A). An E6-specific response to peptide pool E6.4 spanning the last aa (109–158) was detected in five children, of whom two had response also against the pool E6.2 (IDs 1B and 8B) and two against pool E6.3 (IDs 1B and 5B). E7-specific response to pool E7.2 spanning the second half of the E7 protein (aa 43–98) was detected in three out of ten children (IDs 6B, 7B and 8B). All children of mothers who developed incident CIN3 had an HPV16 E2-specific T-cell response, four of whom also responded to E6 and/or E7. One child (ID8B) of HPV16+ CIN2 mother responded to multiple peptide pools of HPV16. Also one child (ID10B) of the HPV16+ CIN1 mother has response to E6.4 peptide pool. Interestingly, both these children were the ones who harbored HPV16 infection in their oral mucosa at baseline.

Three healthy and HPV negative mothers also showed positive LST responses to all peptide pools except for E6.1 and E7.1 pools. Control group of mothers showed responses to peptide pools E2.1 (mean of the SI 25,65, range 22,07-28,57), E2.2 (mean of the SI 14,47, range 4,13-22,72), E6.2 (mean of the SI 4,07, range 0–4,07), E6.3 (mean of the SI 10,68, range 0–10,68), E6.4 (mean of the SI 13,98, range 4,74-23,21), E7.2 (mean of the SI 7,19, range 0–7,19) and MRM (mean of the SI 37,13, range 22,07-48,35). The control group of mothers showed slightly more E6 and E7 responses than the study group and the control group of children showed fewer responses to peptide pools E6 and E7 than the study group (data not shown). One mother showed positive LST responses only for E2.2 peptide pool when two others showed positive responses for both E2.1 and E2.2 peptide pools. In addition to responses for peptide pools of E2, the one mother showed responses also for peptide pools E6.2 and E6.4 and the other mother showed responses also for E6.3, E6.4 and E7.2 peptide pools. Control group of children showed responses only for peptide pools E2.1 (mean of the SI 11,56, range 3,45-21,98), E2.2 (mean of the SI 7,20, range 4,24-11,62), E6.4 (mean of the SI 4,48, range 0–4,48) and MRM (mean of the SI 81,47, range 9,74-171,63). All responded for peptide pools E2.1 and E2.2 and only one child also for peptide pool E6.4 (data not shown).

### Children of mothers with incident CIN3 have higher HPV16-specific cytokine secretion by their T- cells than those of their mothers

**Figure 3:**
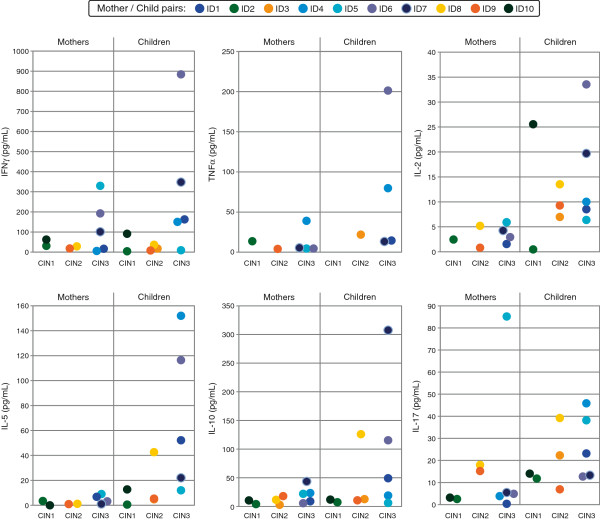
**Cytokines accompanying the proliferative response of T cells in the LST.** The cumulative amounts of the individual cytokine for all peptide pools as determined by CBA are depicted. The mothers and their children are grouped according to the mothers CIN. Only cytokine levels above detection limits are shown. The children of mothers having developed CIN3, showed significantly higher IFN-γ (p = 0.032) and TNF-α (p = 0.008) levels than the other children. The cytokine levels of IL-2 (p = 0.023) and IL-5 (p = 0.028) induced by all peptide pools, were higher among the children than their mothers. In the pair-wise comparison of the mother-child-pairs, the levels of IL-2 and IL-5 showed statistically significant difference between the mother and child (p = 0.013 and p = 0.009, respectively). Control group of mothers showed highest cumulative amounts cytokine levels for IFN-γ (mean 139,03, range 12,99-210,13), IL-17 (mean 60,00, range 3,39-101,04) and IL-10 (mean 40,29, range 7,30-83,24) followed by IL-2 (mean 14,54, range 6,93-29,67), IL-5 (mean 13,65, range 1,65-29,13) and TNF-α (mean 2,88, range 0–6,41) (data not shown).

Figures 2B and 3 summarize the results of cytokines produced by the PBMC in the LST of both the mothers and their children, stratified by the mother’s degree of CIN. Overall, IFN-γ, IL-17A and IL-2 were the most predominant cytokines followed by IL-10, IL-5 and TNF-α in mothers, whereas all cytokines were found among the children. IL-4 was rarely detected and therefore not shown. HPV16–specific, Th1 cytokine (IFN-γ and TNF-α) responses accompanying the proliferative T-cell responses were found in 6/10 mothers and in 5/10 children. This was frequently (in 5/6 mothers and in all children) accompanied by Th2 (IL-5 producing) T-cells. Mother ID3A was the only mother who had no IFN-γ or IL-5 response for any of the peptide pools and showed a proliferative T-cell response only against the pool E2.1 (Figures 2B and 3). She developed an incident CIN2, HPV16 DNA being detectable in her cervical and oral samples twice and four times, respectively. In addition to HPV16, also HPV genotypes 6, 31, 70, 82 (in cervical smear) and 66 (in oral sample) were detected. She also tested constantly HPV6 seropositive.

HPV16 E2-specific IFN-γ producing T-cells to both E2.1 and E2.2 peptides were detected in 7 mothers (Figure 2B). HPV16 E7.1 peptide specific IFN-γ producing T-cells were found in only one of the mothers (ID8A), while E6.2, E6.3 and E6.4 responses were found in three (IDs 6A, 9A, 10A), four (IDs 4A, 6A, 7A and 9A) and four (IDs 2A, 6A, 7A and 10A) mothers, respectively (Figure 2B). Six children had IFN-γ producing Th1 cells against at least one of the peptide pools of HPV16 E2, E6 and E7. The mother of the child ID6B, who had the highest levels of IFN-γ, TNF-α and IL-2, had cervical disease during the entire FU, in which the disease varied from non-squamous intraepithelial lesion (NSIL) to CIN3 with five different HPV genotypes identified.

In 8 out of 10 mothers, T-cells had the capacity to produce IL-2 against E2 protein and 6 of the 10 mothers displayed an IL-2 response to E6. E7.2-induced IL-2 production by T-cells was detected in all three women. HPV16-specific IL-2 responses were more frequently found in children than in their mothers. The concentrations of IL-2 were low, remaining mostly under 24.0 pg/mL except in two children (IDs 6B and 10B). HPV16 induced antigen-specific TNF-α secretion was detected in six mothers and in five children (Figure 2B).

IL-10 and IL-17A were detected in all mothers and children at least as a response to one of the HPV16 E2, E6 or E7 peptides. They were usually detected in parallel, the levels of IL-10 (up to 81.97 pg/mL) being usually higher than those of IL-17A (up to 75.26 pg/mL) for the individual peptide pools (Figure 3). The mother ID5A with the highest IL-17A levels developed CIN3 and tested HPV16 DNA-positive five times without any HPV seropositive response during the FU. Her child had a wider HPV16-specific T-cell proliferative response with positive reaction to four different peptide pools (E2.1, E2.2, E6.3 and E6.4). Interestingly, the highest level of IL-10 was detected in child ID7B, whose mother (ID7A) showed almost an identical history of cervical disease (repeatedly HPV16 DNA-positive and CIN3), similar as mother ID5A described above. However, ID7A was HPV16-seropositive and had also a broader proliferative response against HPV16 than most of other mothers in our study.

Among control group of mothers, the highest cytokine levels were with IFN-γ, IL-17 and IL-10 followed by IL-2, IL-5 and TNF-α. The levels of all cytokines are presented in the legend of the Figure 3. In the control group, the secretion of TNF-α is the most minor comparing to the other cytokines measured (data not shown). In study group, the TNF-α have the fourth highest secreted levels measured, instead.

To summarize, PBMC cultures displaying HPV16-specific proliferative responses produced slightly higher levels of inflammatory cytokines than PBMC cultures not responding by proliferation. Especially Th1 –, Th2 –, and Th17-type cytokines were identified in response to HPV16 peptides (Figure 2B). When comparing the mothers and children as groups the cytokine levels of IL-2 (p = 0.023), and IL-5 (p = 0.028), were higher in children than their mothers for all peptide pools, irrespective of the proliferative status, indicating that they have a more polarized HPV16-specific Th1 and Th2 response. The children of mothers having developed CIN3, showed significantly higher IFN-γ (p = 0.032) and TNF-α (p = 0.008) levels than the other children. In both those cytokine groups mentioned was one outliner, the child with ID6 (Figure 3). In the pair-wise comparison of mother-child-pairs, the levels of IL-2 and IL-5 showed statistically significant difference between the mother and child (p = 0.013 and p = 0.009, respectively).

### HPV16 specific CD4 + CD25 + Foxp3+ regulatory T-cell detection

The frequency of CD4 + CD25 + Foxp3+ cells after 7-day stimulation was analyzed (Figure 2C). Two mothers had positive responses at least to some peptide pools. Mother ID2A showed positivity to peptide pool E2.2, while ID5A showed responses to peptide pools E2.1, E2.2, E6.2, and E7.1. Also in three children, Foxp3+ subsets were expanded: ID6B responded to all pools except pool E2.1; ID9B responded to pools E2.1 and E2.2, and ID10B pool E2.2. Figure 2C represents the percentages of CD4 + CD25 + Foxp3+ regulatory T-cells and CD4 + CD25 + Foxp3 (negative) T-cells for all peptide pools. More Foxp3+ responses were measured among control group of mothers than study group of mothers but the levels were lower. One mother showed no Foxp3+ responses at all, but two other mothers showed responses at least to five different peptide pools including all three peptides (E2, E6 and E7). One mother showed Foxp3+ responses to all HPV peptide pools (data not shown).

## Discussion

In the present study, we examined the HPV16-specific T-cell responses in 10 children born to mothers, who had developed an incident CIN. According to the common dogma no HPV16 specific immune response should be found as the offspring of the CIN mothers had not started their sexual life yet. PBMCs derived from these mother-child pairs were stimulated with HPV16 E2, E6, and E7 peptides to estimate the lymphocyte proliferation response and their cytokine secretion. Our results showed that i) all these children harbored a T-cell response against HPV16 peptides, ii) the responses of the children were broader than those of their mothers (p = 0.0146).

In the present study, the children mounted concomitant T-cell responses to HPV16 E2, E6 and/or E7. Previously we reported that there are highly conserved domains in HPV E2 protein sequence among different HPV genotypes, which could lead to cross-reactivity between the E2 peptides of different types of HPV [13,14]. However, cross-reactive T cells to HPV16 E6 are rare [40] suggesting that the responses observed in these children most likely reflect true circulating HPV16-specific memory T-cells during their early childhood. The authors consider that rather perplexing, because all these children were sexually inexperienced. These results indicate that these children must have had an exposure to HPV16 infection at some body sites. Given that the majority (6/10) of them had detectable HPV DNA in their oral samples, oral mucosa seems to be a plausible route of HPV acquisition and for the stimulation of HPV16-specific adaptive immune responses. These results reinforce the concept that part of HPV infections are acquired already at childhood by non-sexual routes of transmission [21,22] and suggest that HPV-immunity could be evoked early at life. Even in the control group of the HPV-negative mothers an HPV 16 specific immune response was found supporting the view that nearly all women will be infected with HPV. The important question is the age when one will get the first exposure to HPV and how. The individual differences between immune responses of HPV-infected persons will determine either the virus infection will persist or will be resolve such as our results of control groups showed. More studies are needed to discover the crucial differences in the immune systems concerning HPV infections.

Interestingly, rarely an HPV16 E6-specific and no HPV16 E7-specific proliferative T-cell responses were found in the mothers, despite the facts that all had developed an incident CIN as a consequence of a persistent genital HPV16 infection and that the HPV16 E6 and E7 proteins should be expressed in all HPV16–induced CIN3 and CC lesions [41]. Five children displayed T-cell reactivity to E2 and E6, two of whom also reacted to E7. One other child reacted to HPV16 E2 and E7. The children’s response pattern fits with previous data showing that whereas E2- and E6-specific responses are readily found in healthy individuals, this is not the case for E7 [13,37]. The absence of responses in their mothers also is in line with previous data showing lack of responsiveness in patients with CIN and CC [38].

The children’s oral sample tested HPV16-positive in two cases, one was HPV18 positive and one HPV6 positive while two were HPV DNA-negative. Thus, there was no straightforward correlation between the detection of HPV in the children and their HPV-specific immune response, except for the fact that five of the mothers of these six children tested HPV16 and one HPV18 DNA-positive in their cervical samples, and four were seropositive for HPV16 or multiple types.

T helper type 1 cytokine IFN-γ and T helper type 2 cytokine IL-5 production is the characteristic for HPV16-specific immune response among healthy subjects but this is lost in patients with CC [37]. In the present study, the cytokine levels of IL-2, IL-5, IL-10, and IL-17A were higher in children than their mothers indicating that they have a more polarized HPV16-specific Th1 and Th2 response and this would be in line with their disease status. Interestingly, mother’s high-grade CIN3 resulted in the strongest and broadest HPV16-specific CMI response in their children. A worth noticing is that there was one outliner among samples from children of mothers with CIN3, which raised the total level of IFN-γ and TNF-α of those children. Nevertheless, the produced IFN-γ and TNF-α levels were still highest among children of mothers with CIN3 than other children. The one child which has the highest IFN-γ and TNF-α levels, had also highest IL2 and IL-10 levels, so it might be that this child has fairly high HPV-specific immune responses in its entirety.

Many studies have described HPV16–specific CMI reactivity among women with HPV-induced CC or CIN [36-38]. In the present study, all these women had developed incident CIN which was adequately treated, and who consequently could be considered to have passed their HPV-induced disease. However, they cannot be regarded as healthy because of their long-lasting HPV history and increased risk for recurrent disease. In a previous study, increased CD4 + CD25+ Foxp3+ frequency was found in women who developed CIN3 [42]. Thus, increased frequencies of this cell types might predispose to increased risk for high-grade CIN and CC. In the present cohort, two mothers were positive for CD4 + CD25+ Foxp3+ cells, both of them having a persistent HPV16 infection (a known risk factor for recurrent disease). In contrast to all mothers, the children in this study should be considered as healthy and sexually inexperienced subjects. However, three children were already CD4 + CD25 + Foxp3-positive which might indicate that they are at risk for HPV-associated diseases in the future.

## Conclusions

To conclude, our data support the view that HPV infection can be transmitted by non-sexual routes. The overall higher HPV16-specific responses of the children who have not started their sexual life than that of their mothers with CIN suggest that an early transmission may also lead to protective immunity. The exact mechanism remains yet unknown and further studies are urgently needed.

## Abbreviations

CBA: Cytometric bead array; CC: Cervical cancer; CIN: Cervical intraepithelial neoplasia; CMI: Cell-mediated immune; CPM: Counts per minute; CTLs: Cytotoxic T lymphocytes; E: Early protein; Foxp3: Forkhead box P3; FU: Follow up; HPLC: High-performance liquid chromatography; HSIL: High-grade squamous intraepithelial neoplasia; HPV: Human papillomavirus; HR: High-risk; LC: Langerhans cells; LSIL: Low-grade squamous intraepithelial neoplasia; LST: Lymphocyte stimulation test; MRM: Memory response mix; NCIN: Non-cervical intraepithelial neoplasia; NSIL: Non-squamous intraepithelial lesion; SI: Stimulation index; SPPS: Solid phase peptide synthesis; STD: Sexually transmitted disease.

## Competing interests

The authors declare that they have no competing interest.

This study has been supported by the Academy of Finland (16438/2006, #130204/2008), Finnish Cancer Foundation, Sohlberg Foundation, Finnish Dental Society, and the Government Special Foundation (EVO) to Turku University Hospital. The funders had no role in the study design and data analysis, decision to publish, or preparation of the manuscript.

## Authors’ contributions

HMK and AEP performed the research, provided essential reagents and tools. SMS and SEG designed the cohort and the current study. HMK, AEP, SMS, MJPW, SHVDB, and KJS analyzed data and HMK, AEP, SHVDB, MJPW, SMS and KJS contributed in writing the manuscript. All authors read and approved the final manuscript.

## Supplementary Material

Additional file 1: Table S1 Characteristics of the mothers with CIN and their children. The table summarizes the mothers’ medical history of cervical lesions, oral and genital HPV status and HPV-specific serology, as well as the oral HPV DNA status and HPV serology of their children, followed since birth until present. The red color represents HPV16 positive DNA sample, the purple HPV16 seropositive sample, the light green color cervical disease of grade LSIL/NCIN/ASCUS, the yellow color CIN2, the blue color represents CIN3 and the light grey negative sample. A = mother, B = child, baseline = time of the birth, 3d = age of 3 days, 1 mo = age of one month etc. Click here for file

## References

[B1] Smith JS Lindsay L Hoots B Keys J Franceschi S Winer R Clifford GM Human papillomavirus type distribution in invasive cervical cancer and high-grade cervical lesions: a meta-analysis update Int J Cancer 2007 121 621 632 10.1002/ijc.22527 17405118

[B2] Clifford GM Smith JS Aguado T Franceschi S Comparison of HPV type distribution in high-grade cervical lesions and cervical cancer: a meta-analysis Br J Cancer 2003 89 101 105 10.1038/sj.bjc.6601024 12838308 PMC2394204

[B3] Syrjänen K Syrjänen S Epidemiology of human papilloma virus infections and genital neoplasia Scand J Infect Dis Suppl 1990 69 7 17 2175942

[B4] Ostör AG Studies on 200 cases of early squamous cell carcinoma of the cervix Int J Gynecol Pathol 1993 12 193 207 10.1097/00004347-199307000-00001 7688352

[B5] McCredie MR Sharples KJ Paul C Baranyai J Medley G Jones RW Skegg DC Natural history of cervical neoplasia and risk of invasive cancer in women with cervical intraepithelial neoplasia 3: a retrospective cohort study Lancet Oncol 2008 9 425 434 10.1016/S1470-2045(08)70103-7 18407790

[B6] de Sanjosé S Diaz M Castellsagué X Clifford G Bruni L Muñoz N Bosch FX Worldwide prevalence and genotype distribution of cervical human papillomavirus DNA in women with normal cytology: a meta-analysis Lancet Infect Dis 2007 7 453 459 10.1016/S1473-3099(07)70158-5 17597569

[B7] Kanodia S Fahey LM Kast WM Mechanisms used by human papillomaviruses to escape the host immune response Curr Cancer Drug Targets 2007 7 79 89 10.2174/156800907780006869 17305480

[B8] Stanley MA Immune responses to human papilloma viruses Indian J Med Res 2009 130 266 276 19901436

[B9] Frazer IH Interaction of human papillomaviruses with the host immune system: a well evolved relationship Virology 2009 384 410 414 10.1016/j.virol.2008.10.004 18986661

[B10] Scott M Nakagawa M Moscicki AB Cell-mediated immune response to human papillomavirus infection Clin Diagn Lab Immunol 2001 8 209 220 11238198 10.1128/CDLI.8.2.209-220.2001 PMC96039

[B11] Frazer IH Leggatt GR Mattarollo SR Prevention and treatment of papillomavirus-related cancers through immunization Annu Rev Immunol 2011 29 111 138 10.1146/annurev-immunol-031210-101308 21166538

[B12] van der Burg SH Arens R Melief CJ Immunotherapy for persistent viral infections and associated disease Trends Immunol 2011 32 97 103 10.1016/j.it.2010.12.006 21227751

[B13] de Jong A van der Burg SH Kwappenberg KM van der Hulst JM Franken KL Geluk A van Meijgaarden KE Drijfhout JW Kenter G Vermeij P Frequent detection of human papillomavirus 16 E2-specific T-helper immunity in healthy subjects Cancer Res 2002 62 472 479 11809698

[B14] Welters MJ de Jong A van den Eeden SJ van der Hulst JM Kwappenberg KM Hassane S Franken KL Drijfhout JW Fleuren GJ Kenter G Frequent display of human papillomavirus type 16 E6-specific memory t-Helper cells in the healthy population as witness of previous viral encounter Cancer Res 2003 63 636 641 12566307

[B15] Woo YL van den Hende M Sterling JC Coleman N Crawford RA Kwappenberg KM Stanley MA van der Burg SH A prospective study on the natural course of low-grade squamous intraepithelial lesions and the presence of HPV16 E2-, E6- and E7-specific T-cell responses Int J Cancer 2010 126 133 141 10.1002/ijc.24804 19645010

[B16] van Poelgeest MI van Seters M van Beurden M Kwappenberg KM Heijmans-Antonissen C Drijfhout JW Melief CJ Kenter GG Helmerhorst TJ Offringa R van der Burg SH Detection of human papillomavirus (HPV) 16-specific CD4+ T-cell immunity in patients with persistent HPV16-induced vulvar intraepithelial neoplasia in relation to clinical impact of imiquimod treatment Clin Cancer Res 2005 11 5273 5280 10.1158/1078-0432.CCR-05-0616 16033846

[B17] de Vos van Steenwijk PJ Piersma SJ Welters MJ van der Hulst JM Fleuren G Hellebrekers BW Kenter GG van der Burg SH Surgery followed by persistence of high-grade squamous intraepithelial lesions is associated with the induction of a dysfunctional HPV16-specific T-cell response Clin Cancer Res 2008 14 7188 7195 10.1158/1078-0432.CCR-08-0994 19010835

[B18] Mills KH Regulatory T cells: friend or foe in immunity to infection? Nat Rev Immunol 2004 4 841 855 10.1038/nri1485 15516964

[B19] van der Burg SH Piersma SJ de Jong A van der Hulst JM Kwappenberg KM van den Hende M Welters MJ Van Rood JJ Fleuren GJ Melief CJ Association of cervical cancer with the presence of CD4+ regulatory T cells specific for human papillomavirus antigens Proc Natl Acad Sci USA 2007 104 12087 12092 10.1073/pnas.0704672104 17615234 PMC1924590

[B20] Shah W Yan X Jing L Zhou Y Chen H Wang Y A reversed CD4/CD8 ratio of tumor-infiltrating lymphocytes and a high percentage of CD4(+)FOXP3(+) regulatory T cells are significantly associated with clinical outcome in squamous cell carcinoma of the cervix Cell Mol Immunol 2011 8 59 66 21200385 10.1038/cmi.2010.56 PMC4002991

[B21] Merckx M Liesbeth WV Arbyn M Meys J Weyers S Temmerman M Vanden Broeck D Transmission of carcinogenic human papillomavirus types from mother to child: a meta-analysis of published studies Eur J Cancer Prev 2013 22 277 285 10.1097/CEJ.0b013e3283592c46 22990004

[B22] Syrjänen S Current concepts on human papillomavirus infections in children APMIS 2010 118 494 509 10.1111/j.1600-0463.2010.02620.x 20553530

[B23] Smith EM Parker MA Rubenstein LM Haugen TH Hamsikova E Turek LP Evidence for vertical transmission of HPV from mothers to infants Infect Dis Obstet Gynecol 2010 2010 326369 20300545 10.1155/2010/326369 PMC2838362

[B24] Koskimaa HM Waterboer T Pawlita M Grénman S Syrjänen K Syrjänen S Human papillomavirus genotypes present in the oral mucosa of newborns and their concordance with maternal cervical human papillomavirus genotypes J Pediatr 2012 160 837 843 10.1016/j.jpeds.2011.10.027 22137368

[B25] Sarkola M Rintala M Grénman S Syrjänen S Human papillomavirus DNA detected in breast milk Pediatr Infect Dis J 2008 27 557 558 10.1097/INF.0b013e318169ef47 18449059

[B26] Puranen M Yliskoski M Saarikoski S Syrjänen K Syrjänen S Vertical transmission of human papillomavirus from infected mothers to their newborn babies and persistence of the virus in childhood Am J Obstet Gynecol 1996 174 694 699 10.1016/S0002-9378(96)70452-0 8623809

[B27] Cason J Kaye JN Jewers RJ Kambo PK Bible JM Kell B Shergill B Pakarian F Raju KS Best JM Perinatal infection and persistence of human papillomavirus types 16 and 18 in infants J Med Virol 1995 47 209 218 10.1002/jmv.1890470305 8551271

[B28] Castellsagué X Drudis T Cañadas MP Goncé A Ros R Pérez JM Quintana MJ Muñoz J Albero G de Sanjosé S Bosch FX Human Papillomavirus (HPV) infection in pregnant women and mother-to-child transmission of genital HPV genotypes: a prospective study in Spain BMC Infect Dis 2009 9 74 10.1186/1471-2334-9-74 19473489 PMC2696457

[B29] Rintala MA Grénman SE Puranen MH Isolauri E Ekblad U Kero PO Syrjänen SM Transmission of high-risk human papillomavirus (HPV) between parents and infant: a prospective study of HPV in families in Finland J Clin Microbiol 2005 43 376 381 10.1128/JCM.43.1.376-381.2005 15634997 PMC540188

[B30] Rintala MA Grénman SE Järvenkylä ME Syrjänen KJ Syrjänen SM High-risk types of human papillomavirus (HPV) DNA in oral and genital mucosa of infants during their first 3 years of life: experience from the Finnish HPV Family Study Clin Infect Dis 2005 41 1728 1733 10.1086/498114 16288396

[B31] Rintala M Grénman S Puranen M Syrjänen S Natural history of oral papillomavirus infections in spouses: a prospective Finnish HPV Family Study J Clin Virol 2006 35 89 94 10.1016/j.jcv.2005.05.012 16112613

[B32] Rintala MA Louvanto K Rantanen V Grénman SE Syrjänen KJ Syrjänen SM High-risk human papillomavirus associated with incident cervical intraepithelial neoplasia developing in mothers in the Finnish Family HPV Study cohort Scand J Infect Dis 2012 44 115 125 10.3109/00365548.2011.619999 22053923

[B33] Louvanto K Rintala MA Syrjänen KJ Grénman SE Syrjänen SM Genotype-specific persistence of genital human papillomavirus (HPV) infections in women followed for 6 years in the Finnish Family HPV Study J Infect Dis 2010 202 436 444 10.1086/653826 20557239

[B34] Britten CM Janetzki S van der Burg SH Huber C Kalos M Levitsky HI Maecker HT Melief CJ O’Donnell-Tormey J Odunsi K Minimal information about T cell assays: the process of reaching the community of T cell immunologists in cancer and beyond Cancer Immunol Immunother 2011 60 15 22 10.1007/s00262-010-0940-z 21080166 PMC3029829

[B35] Janetzki S Britten CM Kalos M Levitsky HI Maecker HT Melief CJ Old LJ Romero P Hoos A Davis MM “MIATA”-minimal information about T cell assays Immunity 2009 31 527 528 10.1016/j.immuni.2009.09.007 19833080 PMC3762500

[B36] Welters MJ Kenter GG Piersma SJ Vloon AP Löwik MJ Berends-van der Meer DM Drijfhout JW Valentijn AR Wafelman AR Oostendorp J Induction of tumor-specific CD4+ and CD8+ T-cell immunity in cervical cancer patients by a human papillomavirus type 16 E6 and E7 long peptides vaccine Clin Cancer Res 2008 14 178 187 10.1158/1078-0432.CCR-07-1880 18172269

[B37] de Jong A van Poelgeest MI van der Hulst JM Drijfhout JW Fleuren GJ Melief CJ Kenter G Offringa R van der Burg SH Human papillomavirus type 16-positive cervical cancer is associated with impaired CD4+ T-cell immunity against early antigens E2 and E6 Cancer Res 2004 64 5449 5455 10.1158/0008-5472.CAN-04-0831 15289354

[B38] Heusinkveld M Welters MJ van Poelgeest MI van der Hulst JM Melief CJ Fleuren GJ Kenter GG van der Burg SH The detection of circulating human papillomavirus-specific T cells is associated with improved survival of patients with deeply infiltrating tumors Int J Cancer 2011 128 379 389 10.1002/ijc.25361 20473854

[B39] de Vos van Steenwijk PJ Ramwadhdoebe TH Löwik MJ van der Minne CE Berends-van der Meer DM Fathers LM Valentijn AR Oostendorp J Fleuren GJ Hellebrekers BW A placebo-controlled randomized HPV16 synthetic long-peptide vaccination study in women with high-grade cervical squamous intraepithelial lesions Cancer Immunol Immunother 2012 61 1485 1492 10.1007/s00262-012-1292-7 22684521 PMC3427705

[B40] van den Hende M Redeker A Kwappenberg KM Franken KL Drijfhout JW Oostendorp J Valentijn AR Fathers LM Welters MJ Melief CJ Evaluation of immunological cross-reactivity between clade A9 high-risk human papillomavirus types on the basis of E6-Specific CD4+ memory T cell responses J Infect Dis 2010 202 1200 1211 10.1086/656367 20822453

[B41] Bosch FX Lorincz A Muñoz N Meijer CJ Shah KV The causal relation between human papillomavirus and cervical cancer J Clin Pathol 2002 55 244 265 10.1136/jcp.55.4.244 11919208 PMC1769629

[B42] Molling JW de Gruijl TD Glim J Moreno M Rozendaal L Meijer CJ van den Eertwegh AJ Scheper RJ von Blomberg ME Bontkes HJ CD4(+)CD25hi regulatory T-cell frequency correlates with persistence of human papillomavirus type 16 and T helper cell responses in patients with cervical intraepithelial neoplasia Int J Cancer 2007 121 1749 1755 10.1002/ijc.22894 17582606

