# Supplementary material for: Human papillomavirus 16 E2-, E6- and E7-specific T-cell responses in children and their mothers who developed incident cervical intraepithelial neoplasia during a 14-year follow-up of the Finnish Family HPV cohort
Source: J Transl Med. 2014 Feb 13;12:44. doi: 10.1186/1479-5876-12-44 (PMC3929154; doi:10.1186/1479-5876-12-44)
Supplement: Additional file 1: Table S1 — Characteristics of the mothers with CIN and their children. The table summarizes the mothers’ medical history of cervical lesions, oral and genital HPV status and HPV-specific serology, as well as the oral HPV DNA status and HPV serology of their children, followed since birth until present. The red color represents HPV16 positive DNA sample, the purple HPV16 seropositive sample, the light green color cervical disease of grade LSIL/NCIN/ASCUS, the yellow color CIN2, the blue color represents CIN3 and the light grey negative sample. A = mother, B = child, baseline = time of the birth, 3d = age of 3 days, 1 mo = age of one month etc. [file 1479-5876-12-44-S1.pdf]

			baseline	3 d	1 mo	2 mo	6 mo	12 mo	18 mo	24 mo	30 mo	36 mo	42 mo	48 mo	54 mo	60 mo	66 mo	72 mo	78 mo	84 mo	90 mo	96 mo	102mo	108 mo	120 mo	124 mo
ID1	A	Cervical HPV	-			-		16,31		-		16,56														
		Pap/Colp	NSIL					NSIL	NSIL	NSIL		NSIL								NSIL		LSIL	CIN3	CIN1	NSIL	
		Antibodies	6,11,16,18					6,11,16,18		6,11,16		16														
	B	oral HPV	-	-	-	-	-	-		-		-														
		Antibodies				-	-	-		-		-														
ID2	A	Cervical HPV	16,31,42			16,18		31		58	-	16,58									16					
		Pap/Colp	ASCUS			NSIL		ASCUS	NSIL	NSIL	NSIL/CIN1	NSIL/NCIN	NSIL/NCIN	NSIL/NCIN							NSIL	NSIL				
		Antibodies	6,16					16,11,18		6,16		6,18														
	B	oral HPV	31	-	-	-	-	-		-		70						18					31			
		Antibodies								16,18																
ID3	A	Cervical HPV	31			31		16, 70		6, 82		16														
		Pap/Colp	ASCUS					NSIL		NSIL		NSIL									LSIL	CIN2	CIN2	NSIL		
		Antibodies	6					6, 16, 18		6, 18																
	B	oral HPV	31	6	33	-	-	-		-								31								
		Antibodies				6	-	-		-		6														
ID4	A	Cervical HPV	-			-	-	58		18, 31		16, 39						-							31	
		Pap/Colp	NSIL					NSIL		NSIL		NSIL							NSIL		NSIL			HSIL	CIN2	CIN3
		Antibodies	16					6, 16, 18		6, 16		6														
	B	oral HPV	-	-	-	-	-	-		-		-														
		Antibodies			16			-		-		6														
ID5	A	Cervical HPV	-			-		16, 59		16		16								-	16	16				
		Pap/Colp	NSIL					NSIL		NSIL		NSIL						ASCUS	ASCUS		CIN3	CIN3	NCIN	NSIL		
		Antibodies	-					-		-		-														
	B	oral HPV	6																							
		Antibodies				-	-	6		6, 18		16														
ID6	A	Cervical HPV	43			18,31		18			51	51							-					58	58	
		Pap/Colp	NSIL					ASCUS	LSIL, NCIN	NSIL, NCIN	ASCUS	ASCUS	NSIL/NCIN	LSIL/NCIN	ASCUS/NCIN		ASCUS		NSIL	NSIL		NSIL	HSIL	CIN3	CIN3	NCIN
		Antibodies	6,16,18,45					6,16,18,45,11			6,16,18	6,16,18														
	B	oral HPV	-	-	18	-	-	-		-		-														
		Antibodies					6	6		6		6														
ID7	A	Cervical HPV	16			-		16		16								16			16	16				
		Pap/Colp	NSIL					NSIL		NSIL								ASCUS	ASCUS		CIN3	CIN3	NSIL	NSIL		
		Antibodies	16					16		16																
	B	oral HPV	-	-	-	-	-	-		-		-														
		Antibodies			-	-	-	6		6																
ID8	A	Cervical HPV	16			16		16		16		16					16	16								
		Pap/Colp	NSIL					NSIL		NSIL		NSIL				CIN2	NCIN	NCIN/ NSIL	NSIL	NSIL			CIN2			
		Antibodies	-					-		-		-														
	B	oral HPV	16		16	-	-	-		-		-						-								
		Antibodies			-	-	6	6		-		-						-								
ID9	A	Cervical HPV	16,59			16,59												16	16			-				
		Pap/Colp																ASCUS	CIN2	CIN2	CIN1	CIN2	NSIL			
		Antibodies	6																							
	B	oral HPV	-	-	-		-											-								
		Antibodies			6		-																			
ID10	A	Cervical HPV	-			-		-			-	16							16, 56				16, 66	16, 66		
		Pap/Colp	NSIL					NSIL			NSIL	NSIL						NSIL	LSIL	LSIL, NCIN			LSIL/ CIN1	NCIN		
		Antibodies	-					16			-															
	B	oral HPV	16		16	-	-	-			-	-						-								
		Antibodies			-	-		6			-	-														
			baseline	3 d	1 mo	2 mo	6 mo	12 mo	18 mo	24 mo	30 mo	36 mo	42 mo	48 mo	54 mo	60 mo	66 mo	72 mo	78 mo	84 mo	90 mo	96 mo	102mo	108 mo	120 mo	124 mo
